# Supplementary material for: A statistical theory of optimal decision-making in sports betting
Source: PLoS One. 2023 Jun 28;18(6):e0287601. doi: 10.1371/journal.pone.0287601 (PMC10306238; doi:10.1371/journal.pone.0287601)
Supplement: Attachment — Submitted filename: PONE-D-22-34727 - Response to Reviewers PONE-D-22-34727.pdf [file pone.0287601.s001.pdf]

“Statistical Principles of Optimal Decision-Making in Sports Wagering”

Response to Reviewers

I would like to thank the Reviewer for the provided feedback, which I hope has led to an improved manuscript.

Reviewer’s Comments in BLACK

Author’s Response in BLUE

Reviewer #1: Report on “Statistical Principles of Optimal Decision-Making in Sports Wagering”

This paper analyzes the efficiency of betting markets for American professional football games from 2002-2022. The analysis includes bets on the margin of victory (point spread) and on the total number of points scored.

This summary does not accurately describe the subject matter of the paper. The paper is theoretical in nature and is concerned with the statistical conditions of optimal wagering on sports outcomes. To emphasize the intended contribution of the paper, the title has been changed to “*A statistical theory of optimal decision-making in sports wagering*”.

Moreover, the following sentence has been added to the Abstract to clarify the objective of the paper:

“Here the key decisions facing the sports bettor are cast in terms of the probability distribution of the outcome variable and the sportsbook's proposition.”

To further clarify the gap in knowledge that the paper is attempting to fill, the following sentence has also been added to the Abstract:

“*Although considerable effort has been previously devoted to the analysis of sportsbook odds setting and public betting trends, the principles governing optimal wagering have not been presented.*”

Please note that market efficiency is one of the potential *applications* that can be addressed with the theoretical results.

The paper finds that many bins have sample means that are outside the 0.476 – 0.524 range (see Figure 2). It highlights this finding in the abstract: “approximately two-thirds of matches permit a positive expected profit (but only 43% for bets on point total).” Of course, sample means will vary around population means; with 100 binary outcomes, we would expect the difference between the sample mean and population mean to be approximately 0.5(1 –

0.5)/sqrt(100) = 0.05. There is no analysis of whether we can reject a null hypothesis that the population means fall within the 0.476 – 0.524 interval.

Please note that the manuscript does not present sample means, but rather the *quantiles of the sampling distribution*. The question addressed in the paper is whether the sportsbook proposition (i.e., the point spread), which is known to the bettor, falls within these quantiles.

To address the Reviewer's critique, I have added confidence intervals to all quantiles reported in the manuscript. This alleviates the concern of the findings being spuriously generated as a particular property of the sample data. The confidence interval allows us to evaluate the probability that the sportsbook proposition falls in the 0.476-0.524 quantile interval. Confidence intervals have been added to all experimental analyses: Figures 1, 2, 3, 4, and 5 now all depict the sample quantiles in addition to their 95% confidence intervals.

The results of the empirical analysis have been modified by the inclusion of confidence intervals. Consequently, the result questioned by the Reviewer – "approximately two-thirds of matches permit a positive expected profit (but only 43% for bets on point total)" – has been removed from the Abstract and re-stated (much more modestly) in the Results section.

Put another way, in order for a bettor to have earned a positive profit, they would have had to know in advance of the sample period which specific bins would yield outcome probabilities below 0.476 or above 0.524. The statement that "approximately two-thirds of matches permit a positive expected profit" assumes an ability to cherry pick bins after the fact.

The statement has been removed from the manuscript. The addition of the confidence intervals has led to more modest empirical findings.

More broadly, the reviewer seems to have interpreted the analysis of Figures 2-3 as a recommendation of a betting system instead of a demonstration of the paper's theorems. To bring out the intent of the empirical analyses, the Results section has been reworded to better convey the fact that (last paragraph of Introduction):

"*Importantly, it is not an objective of this paper to propose or analyze the utility of any specific predictors ("features") or models.*"

The general patterns in Figure 1, that home underdogs perform better relative to the point spread than home favorites and that outcomes tend to be less extreme than predicted by extreme over/unders, are well-documented in the literature. The literature in this area is quite vast (search "NFL betting" in Google Scholar to see what I mean). I do not find the incremental contribution of this paper to be compelling enough for PLOS-ONE.

I have performed the search query recommended by the Reviewer. The majority of the results were already cited, but I have added two additional citations to the References.

Please note that during the preparation of the manuscript, an extensive literature search was conducted to put the empirical results into the context of the overall literature. The revised Discussion states that the experimental results were consistent with some previous works:

The empirical data presented here hint at this phenomenon, and are in alignment with previous reports of market inefficiencies in the NFL betting market [14, 30, 11, 13, 4]. Namely, the sportsbook point spread was found to slightly overestimate the median margin of victory for some subsets of the data (Fig 2). Indeed, the stratifications showing this trend were home favorites, agreeing with the idea that the sportsbooks are exploiting the public's bias for wagering on the favorite [18].

I would like to clarify that the presented experimental findings served to demonstrate the application of Theorems 1-4. I would argue that the fact that deploying the theorems results in predictions that match previous results appears to validate the theory.

In this context, I should also mention that I find the claim in the abstract that “here it is shown that sports wagering is effectively a problem of quantile estimation, with the median outcome having a crucial role in optimal decision making” to be a bit strange. That the probability of an outcome being above or below a specified point spread or total, and therefore the quantiles of the distribution of the point spread or total, is central to the profitability of sports betting strikes me as completely obvious to everyone.

I have removed the statement. In the revised Abstract, I have attempted to strike a balance between communicating the results to a broad audience while not coming off as superficial to the experts in the field.
