# Supplementary material for: A statistical theory of optimal decision-making in sports betting
Source: PLoS One. 2023 Jun 28;18(6):e0287601. doi: 10.1371/journal.pone.0287601 (PMC10306238; doi:10.1371/journal.pone.0287601)
Supplement: Attachment — Submitted filename: Review PONE-D-22-34727R1.pdf [file pone.0287601.s002.pdf]

Review PONE-D-22-34727R1

Thank you for giving me the opportunity to review this manuscript. I would like to underline that – in my opinion – the manuscript has merit and I am very confident that it will be worth publishing if several revisions are made to the manuscript.

Positive aspects
I will only very briefly mention the positive aspects of the manuscript as this review is intended to put more effort on possibilities for improvement. However, I would like to underline that I really enjoyed reading the manuscript. I like the fact that theoretical considerations are combined with empirical data. Moreover, it is interesting to see a manuscript in the domain of forecasting/sports betting that is able to answer relevant questions without the use of a concrete forecasting model. The theory is explained intuitively and is easy to follow, the results are well-explained and graphically represented. I'd like to express my respect for the effort done by the author with regard to this manuscript. Please see my several critical comments as an effort to further improve the manuscript.

Shortcomings
My major point of criticism is the integration of the present results into the existing literature. Some claims are too strong and overselling the results, moreover in several parts I'd strongly suggest to consider additional relevant literature. Please find more details on this point below

Abstract: I find the claim "the principles governing optimal wagering have not been presented" way too strong. Please be more careful and precise here. Please also find more information on this point below.

p. 1 Introduction l. 5: The author states "important insights into market efficiency". In my mind this is simplifying as the results of market efficiency papers can point into different directions. So I would suggest to be more careful here by stating that market efficiency has been the subject of investigation or more precise by saying what the important insights were. Moreover, the author states three papers from 1968, 1997 and 2004. Below, I have summarised some more recent papers that might be worth considering:

Angelini, G., & De Angelis, L. (2019). Efficiency of online football betting markets. International Journal of Forecasting, 35(2), 712-721.
Bernardo, G., Ruberti, M., & Verona, R. (2019). Semi-strong inefficiency in the fixed odds betting market: Underestimating the positive impact of head coach replacement in the main European soccer leagues. *The Quarterly Review of Economics and Finance*, *71*, 239-246.
Meier, P. F., Flepp, R., & Franck, E. P. (2021). Are sports betting markets semistrong efficient? Evidence from the COVID-19 pandemic. International Journal of Sport Finance, 16(3).

p. 1 Introduction 2nd paragraph: The author claims that literature on "optimal decision-making from the bettor's perspective" is missing. From my point of view, this is overselling the novelty of the current manuscript. While I certainly see what the manuscript adds to the literature, I would like to see acknowledged that decisions from a bettor's perspective have been considered in the literature explicitly and implicitly.

Examples are the well-known Kelly betting strategy for optimal stake sizes going back to

Kelly, J. L. (1956). A new interpretation of information rate. the bell system technical journal, 35(4), 917-926.

or literature focusing on the effects of non-optimal decisions of bettors

Snowberg, E., & Wolfers, J. (2010). Explaining the favorite–long shot bias: Is it risk-love or misperceptions?. Journal of Political Economy, 118(4), 723-746.

Moreover, it is well established in the forecasting literature to use and test several models for deciding on bets such as several stake sizes (e.g. UNIT BET, UNIT WIN, KELLY) in this example:

Hvattum, L. M., & Arntzen, H. (2010). Using ELO ratings for match result prediction in association football. International Journal of forecasting, 26(3), 460-470.

p.3 Theorem 2: I am surprised to not see the following paper cited.

Wunderlich, F., & Memmert, D. (2020). Are betting returns a useful measure of accuracy in (sports) forecasting?. International Journal of Forecasting, 36(2), 713-722.

For example, Theorem 2 is highly related to the area of no profitable bet presented in the aforementioned paper. In general, I see a lot of overlap between the two papers, both analysing betting decisions both from a theoretical point and based on real-world data. The author could also describe how the current manuscript is different from the aforementioned paper, e.g. by using point spreads and by analysing quantiles.

p.8 Bias-variance in sports wagering: I really like the statement that bettors just need their estimation to be on the correct side of the spread, a fact that is often overlooked in profitable forecasting (see also p. 4 last paragraph). You might discuss that the aforementioned paper of Wunderlich & Memmert and the paper of Hubacek & Sir below make similar statements.

Hubáček, O., & Šír, G. (2023). Beating the market with a bad predictive model. International Journal of Forecasting, 39(2), 691-719.

Further points

Title: Why do you use the wording "sports wagering"? As far as I am concerned, this is pretty uncommon in the literature and – unless there is a specific reason that I am not aware of – I would rather expect the wording "sports betting".

Results Problem formulation point spread: You mention the word point spread betting before giving an explanation on how such bets work. You might want to give a very brief explanation on this before, particularly as a lot of literature in this domain is concentrated on European sports betting markets, where point spreads are not that pronounced.

p.2: Please define or introduce Phi_h, Phi_v is before the first usage.

p. 4 Optimal estimation of the margin of victory: It is assumed that the difference between home and away team m and its estimation are independent. I am neither convinced that this is true nor convinced that this is false. But I am a bit sceptical as this is obviously a strong assumption needed for the further proof. Could you discuss this issue and explain in more detail why this assumption is reasonable?

p.5 Optimality in moneyline wagering: At this point, again, I would suggest to add a reference to European betting markets, where bets without spread (i.e. s=0) are the most common bets. This is also reflected in the literature, which (for example in soccer) is highly concentrated on home, draw,

away betting. I would also suggest to state possible differences between (European) home, draw, away and (North American) moneyline betting.

Koopman, S. J., & Lit, R. (2019). Forecasting football match results in national league competitions using score-driven time series models. International Journal of Forecasting, 35(2), 797-809.
Hvattum, L. M., & Arntzen, H. (2010). Using ELO ratings for match result prediction in association football. International Journal of forecasting, 26(3), 460-470.
Constantinou, A. C., & Fenton, N. E. (2013). Determining the level of ability of football teams by dynamic ratings based on the relative discrepancies in scores between adversaries. Journal of Quantitative Analysis in Sports, 9(1), 37-50.
…among many many others

p. 6 Empirical results: I would strongly suggest to state the source of data where you obtained information on > 5.000 matches. Is it data provided by a company or data openly available online? On p. 9 Materials and Methods it appears that the source is the company usually not opening up on the data. However, I would like to see this explained more clearly.

Same paragraph: This paragraph states means and medians from the data. While the manuscript generally correctly underlines the potential difference between mean and median (e.g. due to strongly skewed distributions), the numbers seem to suggest that the real-world distributions are only very weakly skewed and as such mean and median are closely related (e.g. mean 2.19, median 3; mean 44.43, median 44). I would highly like to see this aspect explained and acknowledged.

Same paragraph: The paragraph says that "The standard deviation […] is nearly 7x the mean, indicating the frequent occurrence of outliers ("blowouts"). This claim, in my mind, is at least misleading. While I agree that a high standard deviation indicated frequent blowouts, this has nothing to do with the mean margin of victory which is rather an estimate of home advantage. If the data would show 0.12 + 14.68 instead of 2.19 + 14.68, would this be a sign of even more blowouts???

p. 7 last paragraph: I really like that the author states that (besides correct forecasting) the bookmaker might have other incentives such as risk management (i.e. book balancing). I wonder and I would like to see discussed whether there might be additional incentives of the bookmaker that contradict perfect forecasting. Just as one example, bookmakers might chose higher odds than reasonable for marketing reasons in some specific games. Might bookmakers in spread betting favour completely equal odds over slightly different odds, although not representing their true belief in the probabilities? To be very precise here, this is a true question from my side, i.e. I don't want to express that this is actually the case.

p. 8 The case for quantile regression: The manuscript states "substantial deviation between the mean and median". This seems to be in contradiction to the data presented in the results section (see three points before). Please adjust this statement or give a better explanation on why you think this is the case.

General point: Not related to any specific part of the manuscript, but you might want to discuss differences between American football and other sports. In terms of forecasting and statistical modelling American football is a very specific challenge as it has different possibilities to score (field goal, touchdown, extra point etc.) while other sports usually have only one possibility to score.
