# Supplementary material for: A statistical theory of optimal decision-making in sports betting
Source: PLoS One. 2023 Jun 28;18(6):e0287601. doi: 10.1371/journal.pone.0287601 (PMC10306238; doi:10.1371/journal.pone.0287601)
Supplement: Attachment — Submitted filename: response_to_reviewers_060123.pdf [file pone.0287601.s003.pdf]

Response to Reviewers

Review PONE-D-22-34727R1

The author would like to acknowledge the efforts of the Reviewers, and particularly Fabian Wunderlich for providing many helpful comments and critiques, all of which have been addressed below.

Below the reviewer text is in blue, and my response is in black font.

Abstract: I find the claim "the principles governing optimal wagering have not been presented" way too strong. Please be more careful and precise here. Please also find more information on this point below.

Agreed. I have toned the language down (i.e., the text has been changed from "have not been presented" to "received less focus" [than analysis of sportsbook odds setting and public betting trends]), and have also elaborated on this claim in the Introduction.

p. 1 Introduction l. 5: The author states "important insights into market efficiency". In my mind this is simplifying as the results of market efficiency papers can point into different directions. So I would suggest to be more careful here by stating that market efficiency has been the subject of investigation or more precise by saying what the important insights were. Moreover, the author states three papers from 1968, 1997 and 2004. Below, I have summarised some more recent papers that might be worth considering:

Angelini, G., & De Angelis, L. (2019). Efficiency of online football betting markets. International Journal of Forecasting, 35(2), 712-721.
Bernardo, G., Ruberti, M., & Verona, R. (2019). Semi-strong inefficiency in the fixed odds betting market: Underestimating the positive impact of head coach replacement in the main European soccer leagues. The Quarterly Review of Economics and Finance, 71, 239-246.
Meier, P. F., Flepp, R., & Franck, E. P. (2021). Are sports betting markets semistrong efficient? Evidence from the COVID-19 pandemic. International Journal of Sport Finance, 16(3).

Agreed, and thanks for providing the references. I have modified the text to read:

A topic of obvious relevance to the betting public, and one that has also been the subject of multiple studies, is the efficiency of sports betting markets \cite{pankoff1968market}. While multiple studies have reported evidence for market inefficiencies \cite{gray1997testing, boulier2003predicting, dixon2004value, mchale2011bradley, angelini2019efficiency, bernardo2019semi, meier2021sports }, others have reached the opposite conclusion \cite{pope1989information,kuypers2000information}. The discrepancy may signify that certain, but not all, sports markets exhibit inefficiencies.

p. 1 Introduction 2nd paragraph: The author claims that literature on “optimal decision-making from the bettor’s perspective” is missing. From my point of view, this is overselling the novelty of the current manuscript. While I certainly see what the manuscript adds to the literature, I would like to see acknowledged that decisions from a bettor’s perspective have been considered in the literature explicitly and implicitly.

Examples are the well-known Kelly betting strategy for optimal stake sizes going back to

Kelly, J. L. (1956). A new interpretation of information rate. the bell system technical journal, 35(4), 917-926.

or literature focusing on the effects of non-optimal decisions of bettors

Snowberg, E., & Wolfers, J. (2010). Explaining the favorite–long shot bias: Is it risk-love or misperceptions?. Journal of Political Economy, 118(4), 723-746.

Moreover, it is well established in the forecasting literature to use and test several models for deciding on bets such as several stake sizes (e.g. UNIT BET, UNIT WIN, KELLY) in this example:

Hvattum, L. M., & Arntzen, H. (2010). Using ELO ratings for match result prediction in association football. International Journal of forecasting, 26(3), 460-470.

Agreed. The “gap statement” has been toned down to now read:

Indeed, the decisions made by sportsbooks to set the offered odds and payouts have been previously analyzed \cite{kuypers2000information,levitt2004gambling,cortis2015expected}. On the other hand, arguably less is known about optimality \emph{on the side of the bettor}. The classic paper by Kelly \cite{kelly1956new} provides the theory for optimizing betsize (as a function of the likelihood of winning the bet) and can readily be applied to sports wagering. The Kelly bet sizing procedure and two heuristic bet sizing strategies are evaluated in the work of Hvattum and Arntzen \cite{hvattum2010using}. The work of Snowberg and Wolfers \cite{snowberg2010explaining} provides evidence that the public's exaggerated betting on improbable events may be explained by a model of misperceived probabilities. Wunderlich and Memmert \cite{wunderlich2020betting} analyze the counterintuitive relationship between the accuracy of a forecasting model and its subsequent profitability, showing that the two are not generally monotonic. Despite these prior works,} idealized statistical answers to the critical questions facing the bettor, namely what games to wager on, and on what side to bet, have not been proposed. Similarly, the theoretical limits on wagering accuracy, and under what statistical conditions they may be attained in practice, are unclear.

p.3 Theorem 2: I am surprised to not see the following paper cited.

Wunderlich, F., & Memmert, D. (2020). Are betting returns a useful measure of accuracy in (sports) forecasting?. International Journal of Forecasting, 36(2), 713-722.

For example, Theorem 2 is highly related to the area of no profitable bet presented in the aforementioned paper. In general, I see a lot of overlap between the two papers, both analysing betting decisions both from a theoretical point and based on real-world data. The author could also describe how the current manuscript is different from the aforementioned paper, e.g. by using point spreads and by analysing quantiles.

This is a most relevant paper! Not sure how I missed it, actually. I have cited it in the Introduction as well as when presenting Theorem 2.

(Text added to Introduction)
Wunderlich and Memmert \cite{wunderlich2020betting} analyze the counterintuitive relationship between the accuracy of a forecasting model and its subsequent profitability, showing that the two are not generally monotonic.

(Text added to Theorem 2)
The result of Theorem 2 is reminiscent of the ``area of no profitable bet'' scenario described in \cite{wunderlich2020betting}. Whereas the latter result is presented in terms of outcome probabilities estimated by the bettor and the sportsbook, Theorem 2 here delineates the conditions under which the sportsbook's point spread assures a negative expectation on the bettor's side.

p.8 Bias-variance in sports wagering: I really like the statement that bettors just need their estimation to be on the correct side of the spread, a fact that is often overlooked in profitable forecasting (see also p. 4 last paragraph). You might discuss that the aforementioned paper of Wunderlich & Memmert and the paper of Hubacek & Sir below make similar statements.

Hubáček, O., & Šír, G. (2023). Beating the market with a bad predictive model. International Journal of Forecasting, 39(2), 691-719.

Agreed - I've added the following text to the Discussion:
Variations on this fundamental result have been previously presented in \cite{wunderlich2020betting} and \cite{hubavcek2019exploiting}, which show that suboptimal models -- those that yield estimates that deviate substantially from the true outcome -- are in fact capable of \emph{systematically} generating positive returns.

Further points

Title: Why do you use the wording "sports wagering"? As far as I am concerned, this is pretty uncommon in the literature and – unless there is a specific reason that I am not aware of – I would rather expect the wording "sports betting".

Honestly I was looking for the most “proper” term with this choice. I’ve changed the title to emphasize clarity but still use “wagering” fairly interchangeably with “betting” throughout the text for stylistic reasons.

Results Problem formulation point spread: You mention the word point spread betting before giving an explanation on how such bets work. You might want to give a very brief explanation on this before, particularly as a lot of literature in this domain is concentrated on European sports betting markets, where point spreads are not that pronounced.

I have added the following text to the beginning of the section:
A popular form of sports wagering in North American markets is so-called ``point spread'' betting, where the objective of the bettor is to predict whether the margin of victory will exceed a value proposed by the sportsbook.

p.2: Please define or introduce Phi_h, Phi_v is before the first usage.

Thanks for catching this. I have added the following text:
Denote the payouts when correctly wagering on the home and visiting teams by ϕ_h and ϕ_v, respectively.

p. 4 Optimal estimation of the margin of victory: It is assumed that the difference between home and away team m and its estimation are independent. I am neither convinced that this is true nor convinced that this is false. But I am a bit sceptical as this is obviously a strong assumption needed for the further proof. Could you discuss this issue and explain in more detail why this assumption is reasonable?

Right. So the analysis becomes much more complicated if one has to account for dependencies between the outcome and the estimate. It is certainly not true when integrating across all games (all \thetas). However, for a given match, it would appear to me that the noise around the outcome (weather, player injuries, etc.) is quite removed from the noise around the estimate (sampling noise). Please note also that the estimator noise (from sampling distribution) comes in before the match has even started.

The following statement has been added to the text:
Given that these are temporally non-overlapping sources of variability -- the sources of noise affecting $\hat{m}$ exert influence on the resulting estimate before the sources of noise have begun to exert their influence on the outcome of the match -- it is assumed that, for a given match, m and $\hat{m}$ are independent.

p.5 Optimality in moneyline wagering: At this point, again, I would suggest to add a reference to European betting markets, where bets without spread (i.e. s=0) are the most common bets. This is also reflected in the literature, which (for example in soccer) is highly concentrated on home,

draw, away betting. I would also suggest to state possible differences between (European) home, draw, away and (North American) moneyline betting.

Koopman, S. J., & Lit, R. (2019). Forecasting football match results in national league competitions using score-driven time series models. International Journal of Forecasting, 35(2), 797-809.
Hvattum, L. M., & Arntzen, H. (2010). Using ELO ratings for match result prediction in association football. International Journal of forecasting, 26(3), 460-470.
Constantinou, A. C., & Fenton, N. E. (2013). Determining the level of ability of football teams by dynamic ratings based on the relative discrepancies in scores between adversaries. Journal of Quantitative Analysis in Sports, 9(1), 37-50.
…among many many others

Thanks for suggesting this. I’ve added the following text to the referenced section:

The moneyline wagering considered in this section is a two-alternative bet that is popular in North American sports. In the European betting markets, the most common type of wager is the three-alternative ``Home-Draw-Away'' bet where there is no point spread and the task of the bettor is to forecast one of the three potential outcomes: $m>0$, $m=0$, or $m<0$, each of which are endowed with a payout (see, for example, \cite{hvattum2010using,constantinou2013determining,koopman2019forecasting}). Clearly the the probability $p(m=0)$ will be non-zero in this context. As a result, the methodology here, which models m by a continuous random variable, cannot be straightforwardly applied to the case of the Home-Draw-Away bet. The extension of the present findings to the case of multi-way bets with discrete m is a potential topic of future research.

p. 6 Empirical results: I would strongly suggest to state the source of data where you obtained information on > 5.000 matches. Is it data provided by a company or data openly available online? On p. 9 Materials and Methods it appears that the source is the company usually not opening up on the data. However, I would like to see this explained more clearly.

Right, so it was provided by a company (bettingdata.com). I have put the name of the service into the paper. Sharing the data on the git repository will be tricky, but I will try to speak to the company about this.

Same paragraph: This paragraph states means and medians from the data. While the manuscript generally correctly underlines the potential difference between mean and median (e.g. due to strongly skewed distributions), the numbers seem to suggest that the real-world distributions are only very weakly skewed and as such mean and median are closely related (e.g. mean 2.19, median 3; mean 44.43, median 44). I would highly like to see this aspect explained and acknowledged.

This is a nuanced point: the similarity between mean and median found here stems from the fact that the data is pooled across all matches (all \thetas). The distinction between mean and

median is relevant for a given match, where larger discrepancies are observed (i.e., when conditioning on the point spread, which models the relative difference in strength between home and away teams, the mean and median begin to diverge more). Because I totally understand your confusion and don't want the reader to be similarly confused, I have removed the reported medians from this part of the text (mean and standard deviation remain).

Same paragraph: The paragraph says that "The standard deviation […] is nearly 7x the mean, indicating the frequent occurrence of outliers ("blowouts"). This claim, in my mind, is at least misleading. While I agree that a high standard deviation indicated frequent blowouts, this has nothing to do with the mean margin of victory which is rather an estimate of home advantage. If the data would show 0.12 + 14.68 instead of 2.19 + 14.68, would this be a sign of even more blowouts???

I see your concern. The ratio of standard deviation to mean (or absolute value of the mean) is termed the coefficient of variation that measures "dispersion" (wikipedia).

The application to this case is a bit nuanced because a margin of victory of "0" has a meaning that is not the same as "0" m or "0" kg, for example.

I have reworded the statement and removed the word "blowouts", though I still retain the part of the text that states that the standard deviation is large relative to the mean, which I do think is relevant to the problem at hand:

The standard deviation of the margin of victory is nearly 7x the mean, indicating a high level of dispersion in the margin of victory, perhaps due to the presence of outliers.

p. 7 last paragraph: I really like that the author states that (besides correct forecasting) the bookmaker might have other incentives such as risk management (i.e. book balancing). I wonder and I would like to see discussed whether there might be additional incentives of the bookmaker that contradict perfect forecasting. Just as one example, bookmakers might chose higher odds than reasonable for marketing reasons in some specific games. Might bookmakers in spread betting favour completely equal odds over slightly different odds, although not representing their true belief in the probabilities? To be very precise here, this is a true question from my side, i.e. I don't want to express that this is actually the case.

Right, I've had a similar thought. The sportsbooks here have started to deviate in very small amounts from the nominal "-110" odds and now will move them in increments of 2 (i.e., -108 or -112). I suspect that they must do this because of the discreteness of the margin of victory. I have not added this to the manuscript as it is speculative on my part.

p. 8 The case for quantile regression: The manuscript states "substantial deviation between the mean and median". This seems to be in contradiction to the data presented in the results section (see three points before). Please adjust this statement or give a better explanation on why you think this is the case.

I have rewritten this section to remove any claims to “substantial deviation between the mean and median” being evidenced by this paper. Instead, I have written:

The findings presented here suggest that conventional regression may be a sub-optimal approach to guiding wagering decisions, whose optimality relies on knowledge of the median and other quantiles. The presence of outliers and multi-modal distributions, as may be expected in sports outcomes, increases the deviation between the mean and median of a random variable. In this case, the dependent variable of conventional regression is distinct from the median and thus less relevant to the decision-making of the sports bettor. The significance of this may be exacerbated by the high noise level on the target random variable, and the low ceiling on model accuracy that this imposes.

General point: Not related to any specific part of the manuscript, but you might want to discuss differences between American football and other sports. In terms of forecasting and statistical modelling American football is a very specific challenge as it has different possibilities to score (field goal, touchdown, extra point etc.) while other sports usually have only one possibility to score.

Thanks for the suggestion, which I agree with. I have added a new final section titled “Sport-specific considerations” to the Discussion:

The three types of wagers considered in this work -- point spread, moneyline, and over-under -- are the most popular bet types in North American sports. The empirical analysis employed data from the National Football League (NFL). One unique aspect of American football is its scoring system, in which the points accumulated by each team increase primarily in increments of 3 or 7 points. The structure of the scoring imposes constraints on the distribution of the margin of victory m. For example, in American Football, the distribution of the margin of victory is expected to exhibit local maxima near values such as: $\pm 3, \pm 7, \pm 10$. In the case of games in the National Basketball Association (NBA), the most common margin of victories tend to occur in the 5-10 interval, reflecting the overall higher point totals in basketball and its most common point increments (2 and 3). As a result, the shape and quantiles of the distribution of m may vary qualitatively between the NBA and NFL.

As a final illustrative example of the importance of the quantiles of m, consider the hypothetical scenario of two American football teams playing a match whose parameters θ have been exactly matched three times previously. In those past matches, the outcomes were $m=3$, $m=7$, and $m=28$. In this fictitious example, the median is 7 but the mean is 16. Now imagine that the point spread for the next match has been set to $s=10$ (home team favored to win the match by 10 points). Assuming that one has committed to wagering on the match, the optimal decision is to bet on the visiting team, despite that fact that the average previous outcome exceeds the spread by 6 points.
